# Early Influence of Emotional Scenes on the Encoding of Fearful Expressions With Different Intensities: An Event-Related Potential Study

**DOI:** 10.3389/fnhum.2022.866253

**Published:** 2022-05-11

**Authors:** Sutao Song, Meiyun Wu, Chunliang Feng

**Affiliations:** ^1^School of Information Science and Engineering, Shandong Normal University, Jinan, China; ^2^School of Education and Psychology, University of Jinan, Jinan, China; ^3^State Key Laboratory of Cognitive Neuroscience and Learning, Beijing Normal University, Beijing, China; ^4^Key Laboratory of Brain, Cognition and Education Sciences, Ministry of Education, School of Psychology, Center for Studies of Psychological Application, Guangdong Key Laboratory of Mental Health and Cognitive Science, South China Normal University, Guangzhou, China

**Keywords:** emotional scenes, intensity, fear expression, ERP, N170, P1

## Abstract

Contextual affective information influences the processing of facial expressions at the relatively early stages of face processing, but the effect of the context on the processing of facial expressions with varying intensities remains unclear. In this study, we investigated the influence of emotional scenes (fearful, happy, and neutral) on the processing of fear expressions at different levels of intensity (high, medium, and low) during the early stages of facial recognition using event-related potential (ERP) technology. EEG data were collected while participants performed a fearful facial expression recognition task. The results showed that (1) the recognition of high-intensity fear expression was higher than that of medium- and low-intensity fear expressions. Facial expression recognition was the highest when faces appeared in fearful scenes. (2) Emotional scenes modulated the amplitudes of N170 for fear expressions with different intensities. Specifically, the N170 amplitude, induced by high-intensity fear expressions, was significantly higher than that induced by low-intensity fear expressions when faces appeared in both neutral and fearful scenes. No significant differences were found between the N170 amplitudes induced by high-, medium-, and low-intensity fear expressions when faces appeared in happy scenes. These results suggest that individuals may tend to allocate their attention resources to the processing of face information when the valence between emotional context and expression conflicts i.e., when the conflict is absent (fear scene and fearful faces) or is low (neutral scene and fearful faces).

## Introduction

Accurately identifying facial expressions is important for social interactions in the human. In daily life, however, facial expressions are always presented with specific contextual information. Facial expression recognition is extremely affected by context, such as body postures (Aviezer et al., 2010; Rajhans et al., 2016), emotional voices (Langeslag et al., 2019), self-related sentences (Aguado et al., 2019; Li S. et al., 2019), the task relevance (Mirabella, 2018; Mancini et al., 2020; Mancini et al., 2022), and emotional scenes (Righart and de Gelder, 2006; Righart and Gelder, 2008a,b; Qiang et al., 2015; Bai et al., 2017; Fernández-Martín et al., 2017; Xu et al., 2017). How does the human brain process facial expressions? Many studies primarily used the prototypical, posed facial expressions provided by volunteers at the request of researchers; but, emotional facial expressions typically arise dynamically from a neutral expression, and this expression strengthens or weakens depending on the situation. For example, a smile may transition into a laugh or mild anger may intensify to rage. Therefore, investigating the influence of emotional context on facial expression recognition, at different expression intensities, is of a great importance.

Accordingly, our brains respond quickly to changes in expression intensity (Leleu et al., 2018). Using an electroencephalogram (EEG), an earlier study demonstrated that maximum amplitude of P1 was attained before the participants confirmed seeing “fear image” in a fear detection task. P1 was observed to peak at about 100 ms after the onset of facial expressions in the occipito-temporal visual cortex (Foti et al., 2010; Luo et al., 2010). Empirical evidence shows that threatening faces induce a greater P1 amplitude than neutral faces (Luo et al., 2010; Smith et al., 2013; Xia et al., 2014), which reflects the rapid detection of threatening information and negative processing bias. However, the intensity effect of P1 is rarely studied.

Studies have also reported that the amplitude of the face-specific component, N170, is enhanced by the intensities of the expressions (Leppänen et al., 2007). According to the traditional view, N170 is sensitive to the structural coding of facial expression and insensitive to the emotional content of facial expression (Martin and Amanda, 2002; Holmes et al., 2003). However, Leppänen et al. (2007) investigated the electrocortical responses to fearful and happy emotional expressions at three levels of intensity (50, 100, 150%), but the intensity effect was only observed following exposure to fear expressions. Similarly, in a study of three negative facial expressions, including fear, disgust, and anger, with different intensities ranging from 50 to 150% with increments of 50%, the amplitudes of N170 were reported to significantly increase as the levels of intensity increased (Sprengelmeyer and Jentzsch, 2006). These studies suggest that the enhancement of the intensity-related N170 amplitude may be sensitive to negative faces. In general, studies that have investigated the intensity effect of isolated facial expressions have demonstrated that expressions with clearer meanings are associated with larger ERP amplitudes, i.e., P1 or N170, during the early processing stages of facial expression.

Righart and de Gelder (2006) were the first to provide electrophysiological evidence showing that context impacts expression processing during the early stages, reporting that the modulation effect of context on expression processing was reflected in the face-specific N170 component. Specifically, they demonstrated that faces without any context evoked the largest N170, and faces (especially fearful faces) in fearful scenes elicited a more negative N170 than faces in neutral scenes (Righart and de Gelder, 2006). These results indicate that scenes affect the early encoding of the face. In a subsequent study, Righart and Gelder (2008b) further clarified the modulation effect of scenes on face processing in an explicit expression discrimination task; showing that faces in fearful scenes induce a more negative N170 than faces in happy and neutral scenes, and fearful faces in fearful scenes evoked a more negative N170 than fearful faces in happy scenes. Both studies partially supported the affective congruency effect. Hietanen and Astikainen (2013) reported a clearer affective congruency effect using the emotional priming paradigm. According to the affective congruency effect, the N170 was stronger for happy expressions primed by positive scenes than N170 for happy expressions primed by negative scenes. Additionally, N170 was stronger for sad expressions primed by negative scenes than N170 for sad expressions primed by positive scenes (Hietanen and Astikainen, 2013). The scene pictures selected by Righart and de Gelder (2006) and Righart and Gelder (2008a) had the same emotional content as the facial expression (e.g., fearful scenes and fearful faces); therefore, they were closer in semantic level.

Recently, multiple studies investigated the influence of context on expression processing using expressions with ambiguous emotional meanings, such as neutral expressions, and found that they were rated more positively in positive contexts and more negatively in negative contexts (Wieser et al., 2014; Li S. et al., 2019). ERP and fMRI studies have also provided evidence of the influence of emotional context on the processing of expressions with ambiguous emotional meanings in the temporal process and the activation of specific brain regions (Schwarz et al., 2013; Wieser et al., 2014; Li S. et al., 2019). This indicates that influence of emotional context on facial expression with different intensities may be varied. Recently, a few studies have provided preliminary evidence of the effect of scenes on expression with different intensities at the behavioral level (Tae-Ho et al., 2012; Li S. et al., 2019). They discovered that the recognition of expressions was largely dependent on the information of emotional scenes (i.e., affective congruency) and affected by the personality traits (e.g., anxiety) of the participants. However, the effects of emotional context on expression processing at varying intensities have yet to be identified.

In this study, we investigated the influence of scenes on the recognition of fear expressions with different intensities using ERP. By presenting integrated pictures of fear expressions with varying intensities (high, medium, and low) and scenes with different valences (fear, neutral, and happy), the subjects were required to identify whether the expression presented on the background of the scene was fear or not. The recognition rate and response time to fear expressions were recorded, and the early components, P1 and N170, were analyzed. We hypothesized that (1) the recognition rate of facial expressions would increase with fear intensity, (2) the recognition rate would be the highest in fearful scenes, happy scene would make fear recognition difficult, and (3) the emotional scenes in which the fear expression appeared would affect the early processing stages of fear expression with different intensities. For N170, we hypothesized that the amplitude evoked by fear expression with higher intensity would be more negative and influenced by the scenes. In addition, fear expression evoked more negative N170 than neutral expression, and the amplitude became more negative as expression intensity increased. And the right lateralized effect for the processing of faces would be observed. We also hypothesized that the faces in fearful scenes would evoke a greater P1 than faces in neutral scenes, and smaller P1 than faces in happy scenes.

## Materials and Methods

### Participants

The sample size in this study was estimated using G*power (version 3.1.9.7). The moderate effect *f* = 0.25 (corresponding η*_*p*_*^2^ is about 0.06), which is expected to reach 0.80 statistical test strength (α = 0.05), was used, and the minimum number of planned samples needed was 22 subjects. To prevent possible loss of subjects, 25 physically and mentally healthy undergraduates from the University of Jinan were recruited. Participants were all right-handed with normal or corrected visual acuity. The handedness was assessed by the Edinburgh Handedness Inventory (Oldfield, 1971). In addition, all participants completed the Beck Anxiety Inventory (Beck and Steer, 1993) before the experiment to ensure that participants could maintain emotional stability during tasks. Scores < 30 points (25.56 ± 2.92), indicated that participants had no severe anxiety. Three participants were excluded because of too many artifact trials (<40 trials per condition). For the final sample, the average age was 19.97 years (*SD* = 1.67; 14 females and eight males). Verbal and written informed consents from all participants prior to the experiment were obtained. Participants were informed that they had the right to terminate the experiment at any time. All participants were received a gift for their participation. The experiment lasted for approximately one and a half hours. This study was approved by the ethics committee of the School of Education and Psychology, University of Jinan.

### Stimuli

In total, 60 emotional scene images, including 20 fearful, neutral, and pleasant scenes, were used in the current study. Most of scene images were selected from the International Affective Picture System, and some were obtained from the internet.^1^ Twenty-two participants were recruited to evaluate the valence (1 = very unpleasant, 9 = very pleasant) and arousal (1 = very calm, 9 = extremely arousing) of each emotional scene image using a 9-point scale. In term of valence, the results showed that pleasant scenes scored significantly higher than neutral scenes, which in turns scored significantly higher than fearful scenes (pleasant scenes: *M* = 7.27, *SE* = 0.17, neutral scenes: *M* = 4.88, *SE* = 0.13, fearful scenes: *M* = 2.76, *SE* = 0.23, *ps* < 0.05). In addition, fearful and pleasant scenes were more emotionally arousing than neutral scenes (fearful scenes: *M* = 6.70, *SE* = 0.36, pleasant scenes: *M* = 6.34, *SE* = 0.19, neutral scenes: *M* = 4.46, *SE* = 0.20, *ps* < 0.05). There was no difference in arousal between fearful scenes and pleasant scenes [the scores on valence ratings and arousal rating are presented in Appendix A (Supplementary Material)].

**FIGURE 1:**
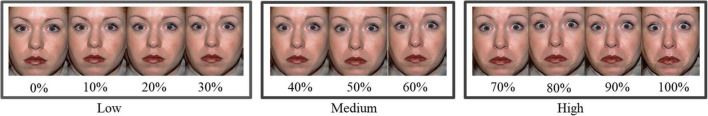
Illustration of fearful facial expressions with different intensities. The image has been obtained from a public database with the publish permission.

Facial expressions were selected from the NimStim database (Tottenham et al., 2009), and 20 pairs of images (fearful and neutral expressions) from 20 individual actors (10 women) were selected to create the different intensities of fearful facial expressions [the actor numbers are presented in Appendix B (Supplementary Material)]. Hair, clothing and other non-face items of the actors were not included. FantaMorphing software^2^ was used to combine the prototypical fearful expressions with the neutral expressions of the same actor. Facial intensities included images ranging from 0 to 100% with 10% increments between consecutive images, and 220 different images depicting 20 different actors with 11 levels of fearful facial expressions were used within each continuum (Figure 1). However, a 10% increment in facial expression intensity is small. Therefore, to evoke a stable ERP waveform, the 11 levels of different intensities were further divided into three groups: low intensity (0, 10, 20, 30%), medium intensity (40, 50, 50, 60%), and high intensity (70, 80, 90, 100%) fearful expressions. A pilot study was conducted to ensure the rationality of this groping.

In the pilot study, ten undergraduate volunteers were asked to indicate if the facial expression was fear or not. The results showed a significant difference among the three intensities; the percentage of recognition of high intense fearful expressions was higher than the percentage of recognition of medium intense fearful expressions, which in turns was higher than the percentage of recognition of low intense fearful expressions (high: *M* = 78.30, *SE* = 2.79, medium: *M* = 34.77, *SE* = 3.19, low: *M* = 10.60, *SE* = 2.63, *ps* < 0.05). To ensure that the number of facial expressions in each group was consistent, the facial expressions with 50% intensity were repeated once so that there were 80 images for low, medium, and high intensity expressions, respectively. All scene and face images were adjusted until they reached a similar luminance, size, and color depth, etc. Facial images were 255*315 pixels and appeared against scene images that were 1.024*768 pixels.

### Procedure

An experiment of 3 (Scene type: fearful, neutral, pleasant) * 3 (Expression intensity: low, medium, high) was designed, and all variables were within-subject factors.

Practice trials were performed to ensure that the participants were familiar with the experimental procedures; these trials were not included in the formal experiment. Overall, there were 720 face-scene compounds: 240 for each group of face intensity, 80 for each experiment condition. For each condition, such as the low-face-intensity and fearful-scene compound condition, the 80 images of facial expressions were shown once, while the 20 scenes were shown four times to produce the compounds. The study was divided into three parts, and participants were given two opportunities to rest. During each part, 240 trials were presented to the subjects, including 80 facial expressions (28 trials for two kinds of intensity and 24 trials for one kind, three intensities balanced in three parts) in 3 emotional scenes. For each condition (low, medium, high), there was an equal number of trials (24 + 28 + 28 = 80 trails). Trials were presented randomly, and the order of the three parts of the experiment was counterbalanced across participants.

**FIGURE 2:**
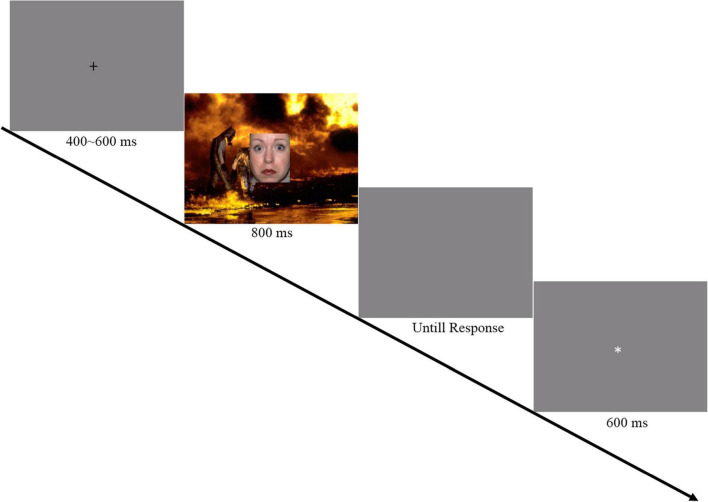
Illustration of the experimental procedure. Fixation mark (*).

During the experiment, participants were seated on a comfortable chair that was located about 70 cm away from the computer screen in an electrically shielded and sound-attenuated room. A single trial was performed as follows: each trial started with a fixation mark (+), which varied randomly between 400 and 600 ms. The face-scene compound was presented for 800 ms, followed by a gray blank screen until the participant pressed the button for a response. Next, a white fixation mark (*) was presented for 600 ms (Figure 2). Participants were instructed to indicate as quickly as possible if the facial expression was fear or not. The experimental design was based on one previous study (Righart and de Gelder, 2006). E-prime 2.0 was used for stimuli presentation and to record behavioral results. The integrated images were presented at the center of screen with a visual angle of 29.94^°^ × 22.45^°^ to scene images and 7.46^°^ × 9.21^°^ to facial expression images.

### Behavioral Data Analysis

The fearful facial expression recognition rate and reaction time were analyzed, and extreme data of reaction time that exceeded three standard deviations were excluded. The proportion of rejected data for each participant was 1.97 ± 0.78% (*M* ± *SD*). A two-way repeated measures ANOVA was conducted using scene type (fearful, neutral, pleasant) and expression intensity (high, medium, low) as the within-subject factors (SPSS v. 17).

#### Electrophysiological Recordings and Analysis

Electroencephalogram data was collected continuously by a 64 Ag/AgCl electrode cap according to the International 10–20 system. All electrodes were referenced online to Cz. Impedance of each electrode was maintained below 10 kΩ during the experiment. Two electrodes were placed approximately 1 cm outside the canthus to record the horizontal electrooculography, and two electrodes were placed about 1 cm above and below the left eye to record the vertical electrooculography (EOG). The EEG and EOG were amplified and digitalized using a Neuroscan Synamp2 (Neuroscan Ltd., Charlotte, NC28269, United States) Amplifier with a band-pass of 0.01–400 Hz and a sampling rate of 1,000 Hz.

Offline, Curry7 (Compumedics Neuroscan United States, Ltd.) was used for data processing. All EEG data were re-referenced to an average reference and filtered with the band pass of 0.1∼30 Hz. Trials with EOG artifacts were corrected using the covariance-based artifact correction algorithms (0 for the Lower threshold, and 200 μV for the Upper one). Afterward, the data contaminated with other artifacts (peak-to-peak deflection exceeding ±100 μV) were excluded from averaging. Next, the EEG recording was segmented into epochs of 1,000 ms starting at 200 ms before stimulus onset. The trials left in each condition were all higher than 60% (low intensity in pleasant: *M* ± *SD* = 69.10 ± 5.53; medium intensity in pleasant: *M* ± *SD* = 67.34 ± 6.26; high intensity in pleasant: *M* ± *SD* = 70.08 ± 2.41; low intensity in neutral: *M* ± *SD* = 66.57 ± 5.28; medium intensity in neutral: *M* ± *SD* = 68.29 ± 6.55; high intensity in neutral: *M* ± *SD* = 71.17 ± 3.37; low intensity in fear: *M* ± *SD* = 63.42 ± 7.89; medium intensity in fear: *M* ± *SD* = 63.33 ± 6.34; high intensity in fear: *M* ± *SD* = 61.86 ± 5.21).

According to previous related studies (Frenkel and Bar-Haim, 2011; Qiang et al., 2015; Malaia et al., 2019), P1 was analyzed for PO3, PO4, O1, and O2 and N170 was analyzed for P7, P8, PO7, and PO8. ERP analyses focused on the peak amplitudes in the following time windows: 60∼140 ms (P1) and 130∼200 ms (N170). All ERP data were analyzed using three-way-repeated measures ANOVA with scene type (fearful, neutral, pleasant), expression intensity (high, medium, low) and hemisphere (left, right) as the within-subject factors. The Greenhouse-Geisser correction was applied where sphericity was violated. When the main effect or an interaction was significant, pairwise comparisons were performed with the Bonferroni correction (SPSS v. 17).

## Results

### Behavioral Data

**FIGURE 3:**
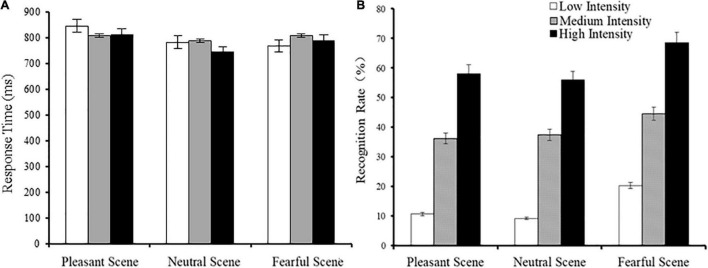
Response time **(A)** and fearful expression recognition rate **(B)** when faces appeared in different scenes (*M* ± *SE*).

There was a significant main effect of fearful expression intensity [*F*_(2,48)_ = 64,14, *p* < 0.001, η*^2^* = 0.73] on the recognition rate. The recognition rate of high intensity fearful expressions was significantly higher than that of medium intensity fearful expressions, and the recognition rate of medium intensity fearful expressions were significantly higher than that of low intensity fearful expressions (high vs. medium: *p* < 0.001; medium vs. low: *p* < 0.001). There was a significant main effect of emotional scenes [*F*_(2,48)_ = 5.57, *p* = 0.006, η*^2^* = 0.19] on the recognition rate, and the *post-hoc* comparison showed that the recognition rate of fearful expressions in fearful scenes was significantly larger than that of neutral scenes (fearful vs. neutral scenes: *p* = 0.019). The recognition rate of fearful expressions in fearful scenes was higher than that of fearful expressions in pleasant scenes; however, this difference was not significant (fearful vs. pleasant scenes: *p* = 0.128). There was no interaction between emotional scene type and facial expression intensity [*F*_(4,96)_ = 1.74, *p* = 0.147, η*^2^* = 0.068]. No differences were found in reaction time (*ps* > 0.05). The reaction time and recognition rate of fearful facial expressions is shown in Figure 3.

### Event-Related Potential Data

#### P1

A significant emotional scene effect [*F*_(2,42)_ = 8.62, *p* < 0.001, η*^2^* = 0.29] was observed on the amplitude of P1. The P1 amplitudes induced by fearful and pleasant scenes were significantly higher than the P1 amplitude induced by neutral scenes (fearful vs. neutral scenes: *p* = 0.003; pleasant vs. neutral scenes: *p* = 0.008). There was no significant main effect of fearful expressions intensities [*F*_(2,42)_ = 0.39, *p* = 0.682, η*^2^* = 0.018] on the P1 amplitude. Neither the hemisphere effect nor the interaction between fearful expression intensities and emotional scenes were significant [*F*_(4,84)_ = 0.42, *p* = 0.796, η*^2^* = 0.019].

#### N170 (130–200 ms)

**FIGURE 4:**
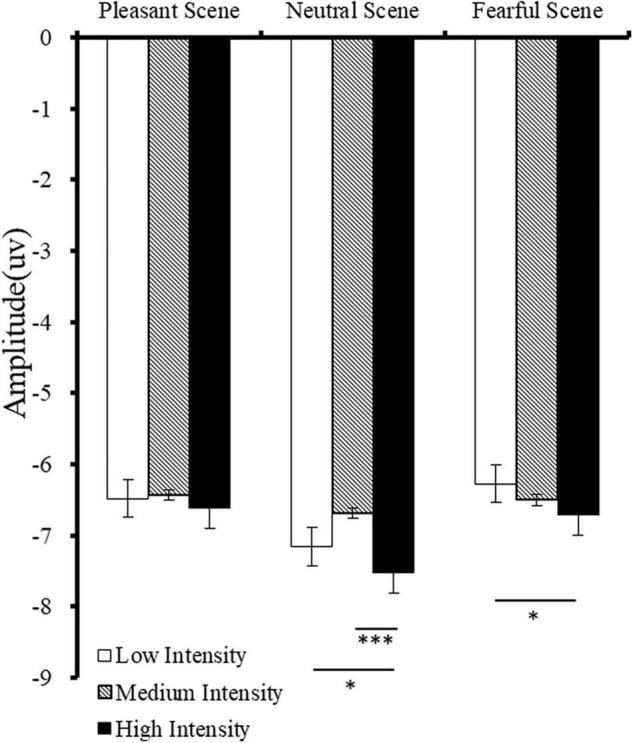
Mean amplitudes of N170 for different fearful facial expression intensities in different scenes. Error bars indicate standard errors, *p < 0.05. ^***^p < 0.001.

A main effect was found for emotional scenes [*F*_(2,42)_ = 11.07, *p* < 0.001, η*^2^* = 0.35] on the amplitude of N170. *Post-hoc* tests confirmed that the amplitude of N170 in neutral scene was significantly higher than the N170 amplitude in pleasant and fearful scenes (neutral vs. pleasant scenes: *p* = 0.007; neutral vs. fearful scenes: *p* = 0.001). There was a significant main intensity effect [*F*_(2,42)_ = 8.05, *p* < 0.001, η*^2^* = 0.30] on the N170 amplitude, and *post-hoc* analyses demonstrated that the N170 amplitude in high intense fearful facial expression was higher than that in medium and low intense fearful facial expressions (high vs. medium: *p* = 0.001; high vs. low: *p* = 0.039) (Figure 4). Finally, there was a significant main effect of hemisphere [*F*_(1,21)_ = 15.93, *p* < 0.001, η*^2^* = 0.43], on the N170 amplitude; the N170 amplitude was higher in the right hemisphere than that in the left hemisphere (right: *M* = –8.30; Left: *M* = –5.12, *p* < 0.001).

**FIGURE 5:**
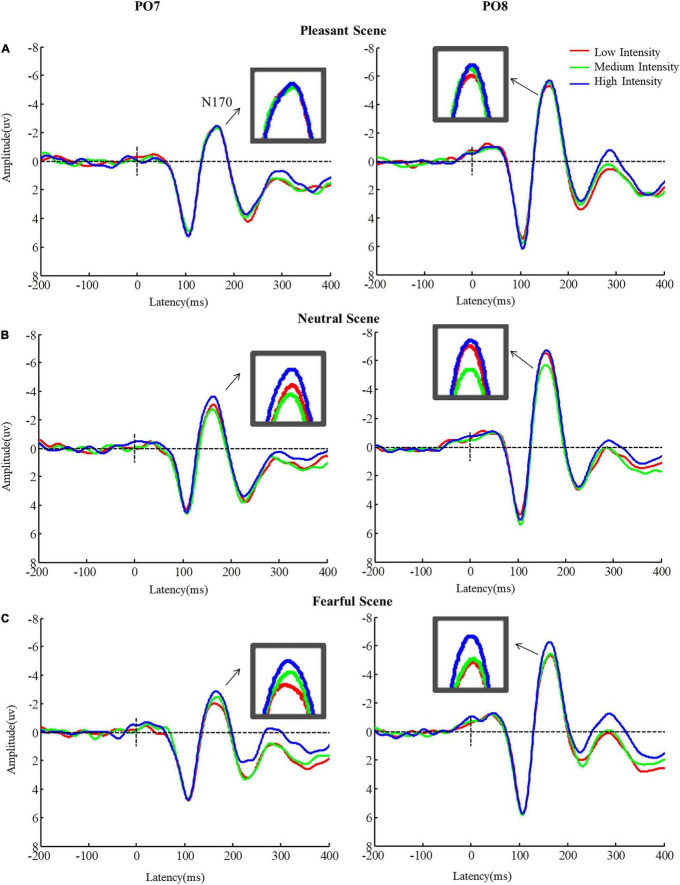
Grand Average N170 amplitudes for **(A)** low intensity, **(B)** medium intensity, and **(C)** high intensity fearful facial expressions in different emotional scenes.

In addition, there was a significant interaction between emotional scenes and fearful facial expression intensities [*F*_(4,84)_ = 3.67, *p* = 0.010, η*^2^* = 0.15] on the N170 amplitude. In neutral scenes, N170 for high intensity fearful facial expressions was higher than that for medium intensity fearful facial expressions (high vs. medium: *p* < 0.001). In fearful scenes, the N170 amplitude for high intensity fearful facial expressions was higher than that for low intensity fearful facial expressions (high vs. low: *p* = 0.026), but no differences were found in the pleasant scenes (*ps* > 0.05). However, for low intense fearful expressions, the N170 amplitude was more negative when faces appeared in neutral scenes than the N170 amplitude when faces appeared in fearful and pleasant scenes (neutral vs. fearful scenes: *p* = 0.002; neutral vs. pleasant scenes: *p* < 0.001). The same result was observed for high intensity fearful expressions (neutral vs. fear scenes: *p* = 0.001; neutral vs. happy cenes: *p* = 0.015), but this result was not significant for medium intensity fearful expressions (Figure 5, *ps* > 0.05).

## Discussion

The current study investigated the influence of scenes on the processing of fear expression with different intensities when faces appeared in different scenes. The behavioral results showed that the recognition rate of fear expressions with high intensity was significantly higher than that for medium and low fear expressions. Additionally, the expression recognition rate was the highest when faces appeared in fearful scenes, indicating that scenes influence the recognition of fear expressions. The ERP results showed that the amplitude of N170 was more negative for faces with high intensity when fearful face appeared in fearful and neutral scenes than the amplitude of N170 for faces with low intensity when fearful faces appeared in fearful and neutral scenes; however, no significant results were observed when faces appeared in happy scenes, suggesting that emotional scenes modulate the processing of facial expressions with different intensities.

At the behavioral level, we found that as the intensity of fear expressions increased, the recognition rate of the expressions also increased, indicating that intensity played an important role in expression recognition (Leppänen et al., 2007; Gao and Maurer, 2010; Chen et al., 2020), which is consistent with the ERP results of this study demonstrating that the N170 amplitude increased with the intensity of fearful expressions. In addition, the context of scenes played an important role in facial expression recognition, as the recognition rate of fearful expressions appearing in fearful scenes was significantly higher than that in neutral scenes, reflecting the affective congruency effect (Righart and Gelder, 2008b). However, there was no interaction between scene and intensity level of fearful expressions, and the recognition rate of medium and low intensity fearful expressions were not affected by the types of scene. Previous studies on expressions with ambiguous emotional meanings (e.g., neutral) found that context affected the perception of expressions, reflected in the ratings of valence and arousal levels; for example, the valence ratings in a positive context were more positive, while those in a negative context were more negative (Wieser et al., 2014). In the current study, the participants were asked to indicate whether the expression was fearful or not, and the response times showed no significant difference among all conditions; therefore, the setting of the task may have been too easy and the embedding of the face stimuli in the context does not appear very realistic/naturalistic, which may explain why no interaction was observed.

In addition, recent studies showed that the emotional content of faces interferes with actions only when task-relevant, i.e., the effect of emotions is context-dependent (Mirabella, 2018; Mancini et al., 2020, 2022). Mirabella (2018) and Mancini et al. (2020) showed that task-irrelevant facial emotional expressions did not influence healthy people’s motor readiness. Mancini et al. (2022) showed that the same effect also occur on inhibitory control, i.e., participants made more commission errors for happy than for fearful expressions only when emotional expressions are task relevant. In our case, the faces were always task relevant, which might reflect the neat result regarding the recognition rate. However, such effect was unlikely to be linked to changes in the N170. These changes occur much earlier than the behavioral responses. In future studies, task-irrelevant conditions (e.g., judge the scene and not the facial expression) should be added to investigate further the effect of emotional scenes on the recognition of expressions intensity.

At the level of ERPs, our results demonstrated that the influence of emotional scene information on fearful expression processing with different intensities was first reflected in the N170 component. In neutral scenes, the N170 amplitude induced by high-intensity fearful expression was stronger than that of medium-and low-intensity fearful expressions. In fearful scenes, the N170 amplitude induced by high-intensity fearful expressions was stronger than that induced by low-intensity fearful expressions. However, no significant difference was found for N170 when faces appeared in happy scenes. Previous studies that have investigated the intensity effect of isolated facial expressions have shown that high-intensity fearful expressions evoked stronger N170 amplitudes (Leppänen et al., 2007). After adding the emotional scene information, we found that the N170 amplitude was modulated by the emotional context. When the emotional context and the expression did not conflict (fearful faces in fearful scenes), or when the conflict between the emotional context and the expression was not too high (fearful faces in neutral scenes), the N170 amplitude differed according to different emotional intensities. When the conflict was highest (fearful faces in happy scenes), the N170 amplitude did not different. A recent study (Chen et al., 2020) showed that N170 amplitude induced by low- and high- intensities fearful expressions on negative scene was greater than the same expressions on the positive scene, which seems to be inconsistent with our findings. The most likely possibility is that facial expressions in our study didn’t be grayscaled for maintaining higher ecological validity, and, therefore, the effect from emotional scenes was weakened. Thus, our results are in line with the characteristics of N170 component, which is more sensitive to facial expression (Bruno, 2014). Additionally, studies have shown that the amplitude of N170 is regulated by attention, and when attention was directed to emotional faces, a larger N170 component was induced (Holmes et al., 2003). Similarly, fMRI studies using the target point detection task have reported that when the target point was presented on the left or right side of the emotional face, the activation of the fusiform gyrus was weakened under the effective clue condition (Brassen et al., 2010). Although some studies have found a significant negative correlation between STS activation and N170 amplitude, source localization analyses have indicated that the fusiform gyrus may be the intracephalic activation region corresponding to N170 (Herrmann et al., 2005), and this may relate to the functional connection between STS and the fusiform gyrus. Therefore, one possible explanation for the results of this study is that the emotional scene information triggered a shift in attention. It has been reported that emotional scenes trigger attention transfer as evidenced by participants paying attention to the emotional scene information when asked to judge the orientation of the stripe stimulus on both sides of an emotional scene (Erthal et al., 2005). Furthermore, the isolated face-induced N170 amplitude was greater than that in the condition when the face appeared in the scene (Righart and de Gelder, 2006), suggesting the presentation of the scene distracted the attention resources for face processing. In the present study, conflicting situations were more likely to trigger more noticeable shifts of attention than consistent and non-conflicting situations, resulting in reduced individual attention to emotional faces; therefore, no intensity effects of expressions were observed. Although not explicitly reported in the study by Righart and Gelder (2008a) it was found that the conflicting situation (fearful face presented in a happy scene) induced the smallest N170 (Righart and Gelder, 2008a).

From the level of the individual experimental condition (Figure 4), the high-intensity fearful expressions evoked the largest amplitude of N170 when faces appeared in neutral scenes. Suggesting that the fearful scenes cause a more obvious attention transfer than neutral scenes and consequently does not reflect the affective congruency effect reported in previous studies (Righart and Gelder, 2008b; Hietanen and Astikainen, 2013). Additionally, when the emotional priming paradigm was used, the affective congruency effect reflected in the N170 was reported steadily (Hietanen and Astikainen, 2013). The processing of both positive and negative expressions showed the affective congruency effect; this was probably because in the priming paradigm, the primed scenes are processed first, allowing subjects to pay more attention to the subsequently presented faces. The possibility of attention transfer was small, indicating that attention transfer may play a more important role under the experimental condition when expressions were embedded in the background of scenes. In future studies, emotional scene priming and point detection paradigms could be combined (Yang et al., 2012) to further clarify the role of attention in the process of scene influence on expression processing, and the relationship between intensity of facial expression and exposure duration in the perception of complex scenes (Shakespeare et al., 2014) need to be clarified. In the current study, P1 induced by fearful and pleasurable scenes were significantly higher than that induced by neutral scenes; however, the main effect of face intensity was not significant, indicating that P1 reflects the rapid detection of threatening or evolutionary significant stimuli, and intensive-related information may be further encoded by the later N170 component (Luo et al., 2010).

The current study examined the influence of scenes on fearful expressions with different intensities on the early stage of face processing and partially demonstrated the affective congruency effect–that is, when fearful faces appeared in fearful scenes, the intensity effect was significant. Additionally, our results also revealed that the intensity effect was significant when fearful faces were presented in neutral scenes and partially in fear scenes. Differently, the intensity effect was not significant when the fearful faces were presented in a pleasant context, indicating that the participants paid less attention to the face in the conflicted situation.

While our study highlights several important findings, there are limitations that need to be addressed. First, the study focused on fearful expressions, and the division level of fearful expressions was a gradual change from neutral to fearful. To keep the number of each facial intensity consistency, one level in medium was represented twice as much than the other level. Therefore, the conditions are not exactly comparable. In addition, as there was no positive expression, it was impossible to prove whether the scene had the emotional suppression effect on positive expressions of different intensities in the early processing stage of facial recognition. Second, the fearful expressions selected in this study were all closed-mouth versions. High-intensity fearful expressions in real life are more likely to be accompanied by mouth opening and tooth exposure. Expression with an open mouth elicits a stronger emotional feeling, and the expressions with exposed teeth evoke a greater N170 amplitude, indicating that low levels of visual features have an important impact on the processing of emotional expressions (Dasilva et al., 2016). Furthermore, negative (e.g., angry) open-mouth expressions induce greater early posterior negativity (EPN) than closed-mouth expressions, indicating a higher degree of automatic capture of early attention resources (Langeslag et al., 2018). Therefore, using fearful expressions with natural open mouths may improve the ecological validity of this study. Finally, in addition to scene information from outside of the facial expressions, information of the expression observer, such as personality traits and emotional states, may also affect the processing of fear expressions with different intensities (Wieser and Brosch, 2012). For example, people with high anxiety show a negative bias toward fear expressions (Torrence and Troup, 2018). Thus, it is necessary to further examine the impact of the internal information of the expression observer.

## Conclusion

This study investigated the influence of emotional context information on fearful facial expression recognition with different intensities. The two major conclusions are: (1) As a function of fear facial expression intensity, the accuracy of facial recognition was enhanced, where fear scenes increase the recognition of fear expressions; (2) the early coding of fearful expressions with different intensities was affected by emotional scenes. Under the valence conflict condition between scenes and expressions, individuals do not need to allocate their attention to face processing probably because of a pop out effect.

## Data Availability Statement

The raw data supporting the conclusions of this article will be made available by the authors, without undue reservation.

## Ethics Statement

The studies involving human participants were reviewed and approved by Ethical Committee of University of Jinan. The participants provided their written informed consent to participate in this study.

## Author Contributions

SS and CF conceived and designed the study. SS and MW prepared the manuscript, conducted the experiments, and analysed the data. CF reviewed the manuscript. All authors contributed to the article and approved the submitted version.

## Conflict of Interest

The authors declare that the research was conducted in the absence of any commercial or financial relationships that could be construed as a potential conflict of interest.

## Publisher’s Note

All claims expressed in this article are solely those of the authors and do not necessarily represent those of their affiliated organizations, or those of the publisher, the editors and the reviewers. Any product that may be evaluated in this article, or claim that may be made by its manufacturer, is not guaranteed or endorsed by the publisher.

## Footnotes

1
https://image.baidu.com/

2
http://www.fantamorph.com

## Abbreviations

ERP: event-related potential
EEG: electroencephalogram
fMRI: functional magnetic resonance imaging
STS: superior temporal sulcus
rSTS: right superior temporal sulcus
LPP: late positive potential
EOG: electrooculography
EPN: early posterior negativity.
