# Supplementary material for: Early Influence of Emotional Scenes on the Encoding of Fearful Expressions With Different Intensities: An Event-Related Potential Study
Source: Front Hum Neurosci. 2022 May 11;16:866253. doi: 10.3389/fnhum.2022.866253 (PMC9150066; doi:10.3389/fnhum.2022.866253)
Supplement: Supplementary file 1 [file Table_1.DOCX]

Supplementary Material

**Appendix A**

Table 1. The mean valence and arousal for emotional scenes

| **Pleasant scenes** | | | **Fearful scenes** | | | **Neutral scenes** | | |
| --- | --- | --- | --- | --- | --- | --- | --- | --- |
| **Picture #** | valence | arousal | Picture # | valence | arousal | Picture # | valence | arousal |
| **1000002** | 7.77 | 5.77 | 2000001 | 1.64 | 6.95 | 5390 | 6.05 | 4.77 |
| **1000006** | 7.55 | 6.59 | 2000002 | 2.18 | 6.82 | 7002 | 4.68 | 4.09 |
| **1000013** | 7.73 | 6.73 | 2000004 | 3.27 | 6.05 | 7006 | 5.05 | 4.59 |
| **1000026** | 6.91 | 6.00 | 2000006 | 2.68 | 6.41 | 7009 | 4.86 | 4.18 |
| **1000032** | 7.95 | 6.23 | 2000008 | 2.95 | 6.77 | 7010 | 4.82 | 4.68 |
| **1000033** | 7.77 | 6.64 | 1040 | 1.91 | 6.64 | 7025 | 4.73 | 4.55 |
| **1000041** | 7.68 | 7.09 | 1050 | 2.32 | 6.82 | 7031 | 4.14 | 4.23 |
| **1000061** | 7.91 | 6.64 | 1052 | 2.36 | 7.00 | 7035 | 4.91 | 4.18 |
| **1604** | 6.73 | 5.27 | 1113 | 2.45 | 6.50 | 7060 | 3.95 | 4.45 |
| **4599** | 7.23 | 6.09 | 1120 | 2.27 | 6.95 | 7096 | 4.91 | 4.59 |
| **5628** | 5.86 | 5.77 | 1390 | 4.00 | 6.18 | 7100 | 5.00 | 4.50 |
| **5830** | 7.23 | 6.18 | 1930 | 3.18 | 6.64 | 7175 | 5.09 | 4.64 |
| **8500** | 7.27 | 6.55 | 6190 | 3.86 | 6.18 | 7180 | 5.00 | 5.00 |
| **8501** | 7.00 | 6.86 | 6300 | 2.86 | 6.68 | 7211 | 4.91 | 4.50 |
| **8021** | 6.73 | 6.50 | 6370 | 2.41 | 6.64 | 7217 | 4.55 | 4.36 |
| **8031** | 6.41 | 6.27 | 6510 | 3.23 | 7.09 | 7224 | 4.50 | 4.45 |
| **7502** | 7.55 | 6.50 | 6550 | 2.64 | 7.55 | 7235 | 5.00 | 3.86 |
| **7230** | 7.23 | 6.73 | 8480 | 3.23 | 7.23 | 7545 | 5.91 | 4.68 |
| **5831** | 7.41 | 6.14 | 9594 | 3.27 | 6.18 | 7705 | 5.09 | 4.59 |
| **8210** | 6.91 | 6.27 | 9910 | 2.45 | 6.64 | 7950 | 4.59 | 4.36 |

Note: The picture with 7 digit numbers came from the internet, the rest of the pictures came from the International Affective Picture System (IAPS).

**Appendix B**

The actor number from Nimstim set: 01, 05, 06, 07, 08, 09, 10,11,15,18, 20, 22, 27, 28, 32, 33, 34, 35, 36, 45
